# Endoscopic features and associated histology of an basaloid squamous cell carcinoma of the esophagus at the very early stage

**DOI:** 10.1186/s12885-019-5749-3

**Published:** 2019-05-29

**Authors:** Lianjun Di, Kuang-I Fu, Xinglong Wu, Xuemei Liu, Rui Xie, Rong Zhu, Biguang Tuo

**Affiliations:** 1 Department of Gastroenterology, Affiliated Hospital, Zunyi Medical University, Zunyi, 563003 China; 2 Department of Endoscopy, Kanma Memorial Hospital, Tokyo, Japan; 3 Department of pathology, Affiliated Hospital, Zunyi Medical University, Zunyi, China

**Keywords:** Basaloid squamous cell carcinoma, Early diagnosis, Endoscopic feature, Esophagus

## Abstract

**Background:**

Basaloid squamous cell carcinoma of the esophagus (BSCCE) is generally detected at advanced stage and the prognosis is poorer than advanced conventional esophageal squamous cell carcinoma. Therefore, early detection is a critical to improve patients’ survival. However, only a few cases of early BSCCE have been reported and the endoscopic features of early BSCCE are not well described. We herein report the endoscopic features and associated histology of an early BSCCE limited within the mucosal lamina propria (m2). To our knowledge, this is the earliest BSCCE reported to date.

**Case presentation:**

A 62-year-old male patient was admitted to our hospital because of epigastric pain for 3 months. White light endoscopy revealed a flat lesion with mild sloping at the periphery. The lesion was covered with leukoplakia, and normal vascular network could not be seen in the lesion. Magnifying endoscopy with narrow-band imaging showed abnormal intra-papillary capillary loop categorized as type B1 according to the classification of the Japan Esophageal Society. Iodine staining revealed a less-stained lesion. The lesion was completely resected through endoscopic submucosal dissection. Histopathologically, tumor cells, which were limited within the mucosal lamina propria, formed solid nests and lobule structures, with ribbon-like arrangement of sparse cytoplasm and round to ovoid hyperchromatic nuclei. A final diagnosis of early BSCCE was established.

**Conclusions:**

This is the earliest BSCCE reported to date. The prominent lesion with a gentle rising slope and less-staining or abnormal stain might be initial feature of early BSCCE.

## Background

Basaloid squamous cell carcinoma of the esophagus (BSCCE) is an extremely rare tumor, accounting for only 0.068–4.0% of total esophageal malignant tumors [1–3]. Advanced BSCCE has a poorer prognosis compared to typical squamous cell carcinoma, but the prognosis of early BSCCE does not differ significantly from that of typical squamous cell carcinoma [4–6]. Therefore, early detection is a critical to improve the prognosis of BSCCE. However, early BSCCE is rarely found, and the endoscopic features of early BSCCE are not well described. We herein report the endoscopic features and associated histology of an early BSCCE limited within the mucosal lamina propria which contributes to knowledge of early BSCCE.

## Case presentation

**Fig. 1:**
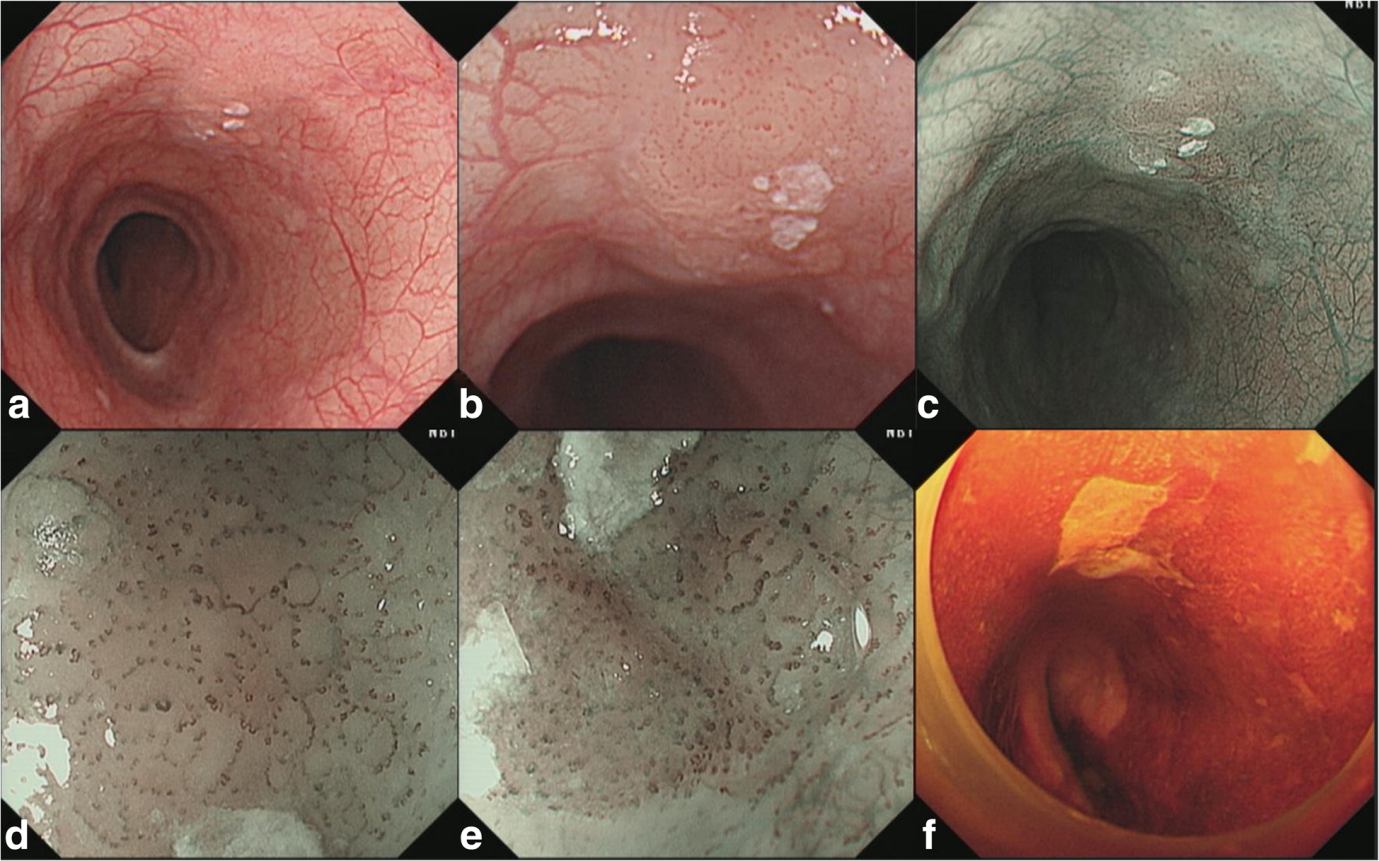
Endoscopic features of superficial basaloid squamous cell carcinoma in the middle esophagus. **a** and **b** White-light endoscopy showing a slight elevated lesion of 5 mm in diameter with some scattered leukoplakia in the surface and disappeared vascular branch network in the lesion. **c** NBI showing an indistinct brownish area. **d** and **e** Magnifying view of ME-NBI showing intrapapillary capillary loops with small-sized avascular area in the lesion. **f** A less-stained lesion under iodine staining

**Fig. 2:**
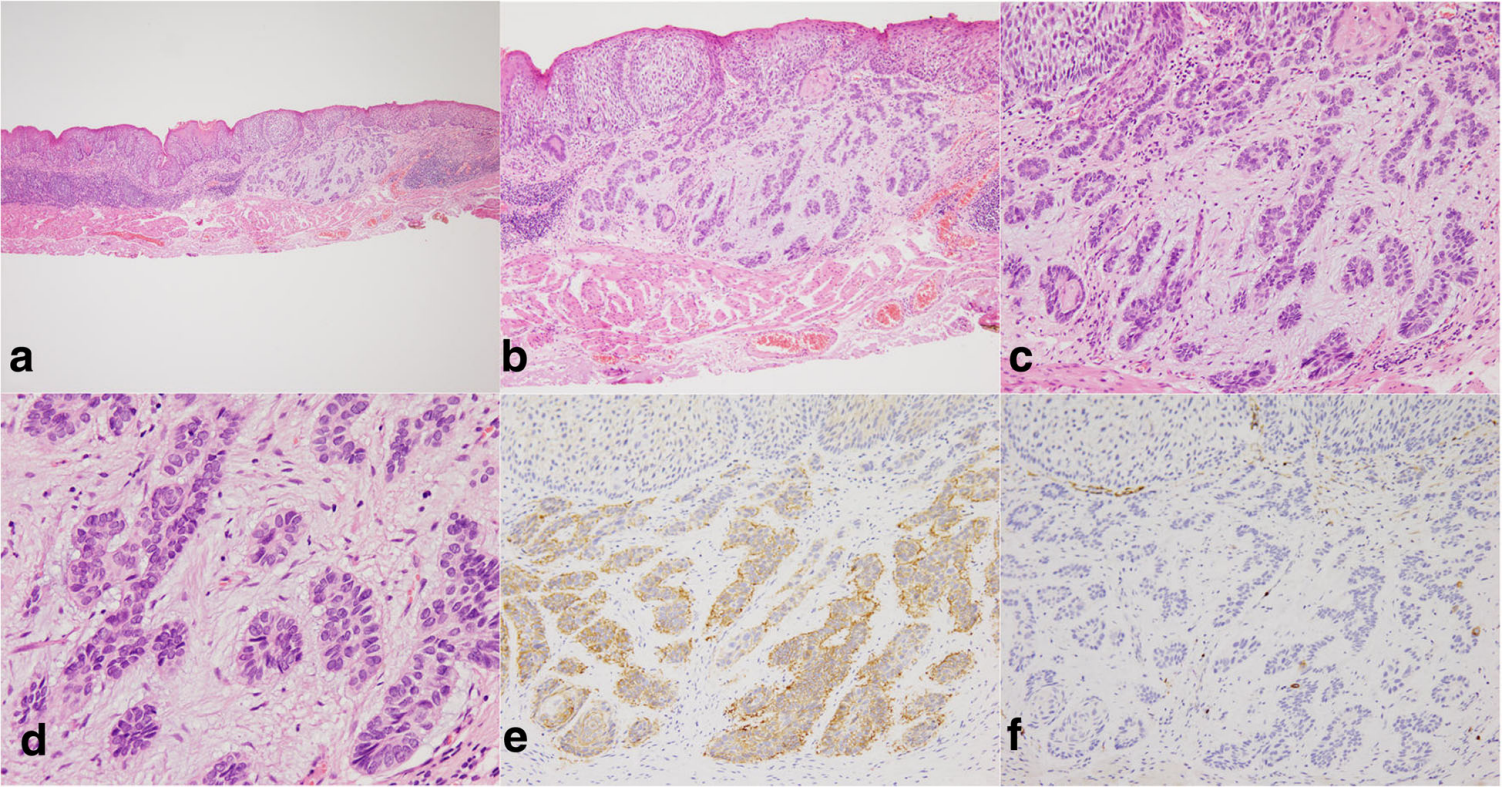
Pathological features of the esophageal basaloid squamous cell carcinoma by hematoxylin & eosin (HE) and immunohistochemical staining (IHC). **a**, **b**, **c**, and **d** There were basaloid hyperchromatic proliferated cancer cells within the mucosal lamina propria. The cancer cells formed solid nests and lobules structures with ribbon-like arrangement of sparse cytoplasm and round to ovoid hyperchromatic nuclei. **e** and **f** Basaloid squamous cancer cells were diffuse positive for Ber-EP4 (**e**) and were negative for CgA (**f**)

**Fig. 3:**
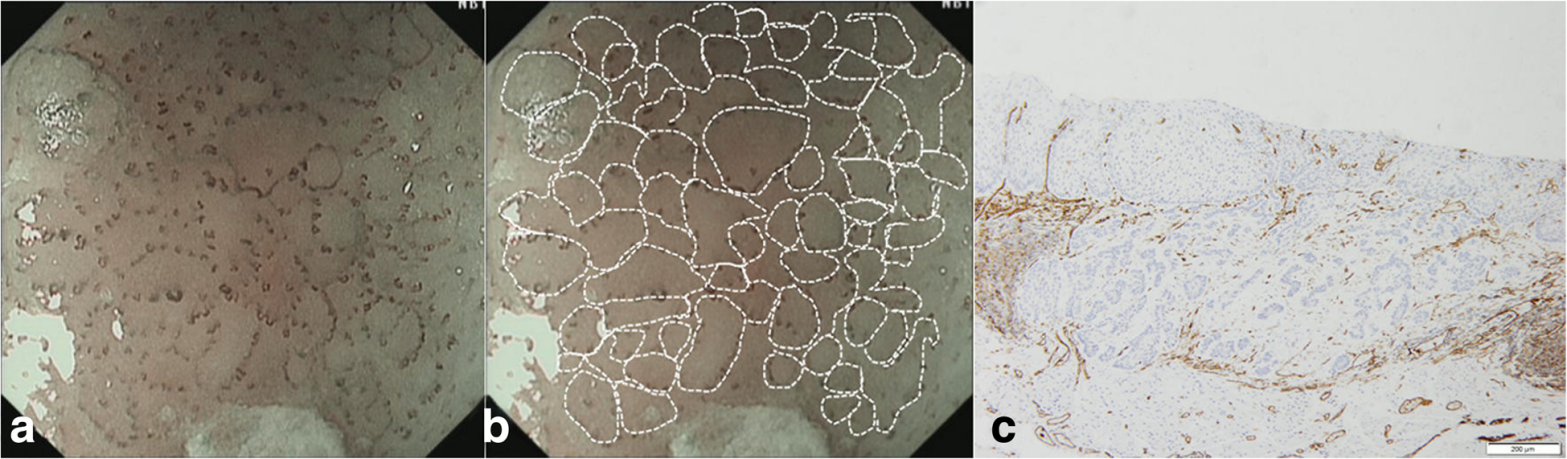
Contrastive analysis for ME-NBI imaging and histopathologic examination of the lesion. **a** and **b** Intrapapillary capillary loops with small-sized avascular area by ME-NBI. **c** The vessels were histopathologically observed around cancer nests by CD31 staining

**Fig. 4:**
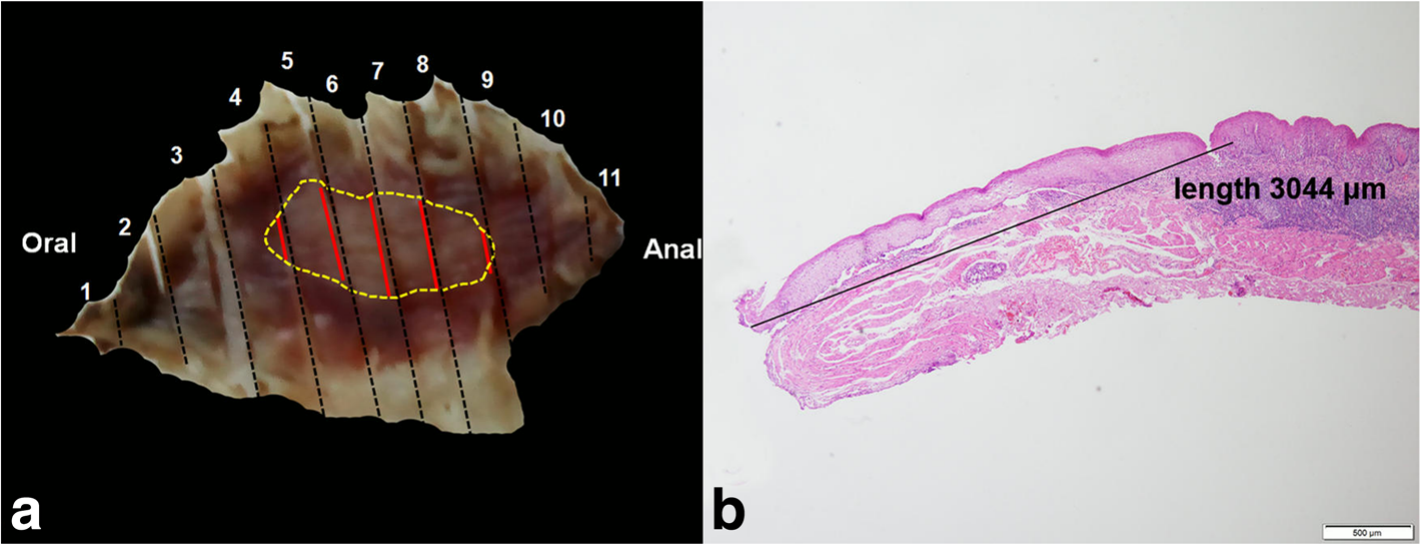
Contrastive analysis for the resected specimen and histopathologic examination. **a** The resected specimen was cut into slices at each 2 mm width. The red lines represent lesion areas in each slice. Oral is oral margin of the specimen. Anal is anal margin of the specimen. **b** Histopathology showing the distance of the lesion to the closest margin of the resected specimen

A 62-year-old male patient was admitted to our hospital because of epigastric pain for 3 months. Esophagogastroduodenoscopy was performed and a superficial esophageal lesion was found in the middle esophagus. White light endoscopy revealed a flat lesion with a gentle rising slope at the periphery of the lesion. There were scattered leukoplakia in the surface of the lesion and normal vascular network could not be seen in the lesion (Fig. 1a, b). Narrow-band imaging (NBI) under endoscopy revealed that the lesion exhibited an indistinct brownish area (Fig. 1c). Magnifying endoscopy with NBI (ME-NBI) showed that there were abnormal intra-papillary capillary loops with small-sized avascular area in the lesion which was then classified as type B1 according to the classification of the Japan Esophagus Society (JES classification) [7] (Fig. 1d, e). Iodine staining (1%) revealed a less-stained lesion (Fig. 1f). A histopathological diagnosis of squamous cell carcinoma was obtained by an endoscopic biopsy. Endoscopic submucosal dissection (ESD) for the lesion was performed. Histopathological examination showed that the component of squamous cell carcinoma was seen in the superficial section of the mucosa. The surface of squamous cell carcinoma was covered by a small amount of non-neoplastic squamous epithelium. Meanwhile, basaloid hyperchromatic proliferated tumor cells were seen mainly in the mucosal lamina propria. The tumor cells formed solid nests and lobule structures with ribbon-like arrangement of sparse cytoplasm and round to ovoid hyperchromatic nuclei cells (Fig. 2a, b, c, d). Immunohistochemically, Ber-EP4 which is a monoclonal antibody to epithelial cells and a sensitive marker of basal cell carcinoma [8] was positive. Chromogranin A which is a well-established marker of gastrointestinal neuroendocrine neoplasms [9] was negative (Fig. 2e, f). In addition, some squamous cell cancer cell nests with globose expansile characteristics growing underneath the non-neoplastic squamous epithelium were found. Abnormal intrapapillary capillary loops were observed around the solid cancer cell nests (Fig. 3a, b). The lesion was histopathologically confirmed as BSCCE limited within the mucosal lamina propria and completely resected. There were no lymphovascular and neural invasions of cancer cells, and no cancer cells to be seen in the lateral and vertical margin of the resected specimen (the distance of the lesion to the closest margin of the resected specimen was 3.044 mm (Fig. 4).

## Discussion and conclusions

Basaloid squamous cell carcinoma commonly occurs in the upper aerodigestive tract, especially in larynx, hypopharynx, and base of the tongue [10, 11]. BSCCE is uncommon and has aggressive biological behavior and poorer prognosis than the more common esophageal squamous cell carcinoma [4–6]. Early detection and diagnosis is important for improving the prognosis of BSSCE.

Macroscopically, BSCCE was mainly visualized as an elevated lesion or submucosal tumor-like structure with normal epithelium on the surface layer which is not significantly different from other special types of esophageal cancer, including adenosquamous carcinoma, small cell carcinoma, and adenoid cystic carcinoma [12]. Therefore, it is considered to be difficult to identify this type of cancer by conventional endoscopy and biopsy, and early stage of BSCCE is more difficultly found. Komatsu et al [13] reported a small BSCCE with invasion of muscularis mucosa in 2001, but the lesion had not been detected by preoperative examinations and there was no description for endoscopic appearance of the lesion. In 2014, Kai et al [14] reported a case of early BSCCE and described the features of the lesion under conventional white light endoscopy and ME-NBI. Histopathological examination showed that the lesion invaded the submucosa. In our case, the lesion was only confined to the mucosal lamina propria by histopathology. To our knowledge, this is the earliest BSCCE reported to date.

The report of Kai et al [14] showed that the lesion presented as a reddish depressed lesion by white light endoscopy. ME-NBI showed irregular loop-shaped microvessels coexisting with thick irregularly branched non-looped vessels. Iodine staining showed a pale brown lesion. The investigators believe that the irregular loop-shaped microvessels coexisting with irregularly branched thick non-looped vessels might be characteristics of early BSCCE. In the present case, we found some different white light endoscopic and magnifying endoscopic features from the report of Kai et al. White light endoscopy revealed a flat lesion with slight rising slope at the periphery of the lesion, indicating the lesion has the morphology of submucosal tumor-like feature which was reported as endoscopic feature of BSCCE [12]. In addition, there were scattered leukoplakia in the surface of the lesion and normal vascular network could not be seen in the lesion. Iodine staining revealed a less-stained lesion which is different from pink color sign of typical squamous cell carcinoma. ME-NBI showed intrapapillary capillary loops with small-sized avascular area. Histopathologically, the intrapapillary capillary loops were observed around solid cancer cell nests with globose expansile characteristics growing underneath the non-neoplastic squamous epithelium, suggesting that the intrapapillary capillary loops observed by ME-NBI correspond to histopathologic results. Small avascular areas observed by ME-NBI are related to solid globose cancer cell nests on histopathology. These demonstrate that endoscopic appearance of early BSCCE might be various. Because it is only a case report, an accumulation of studies involving more patients is necessary to clarify the common features of early BSCCE in endoscopy. We must carefully examine and pay more attention for slight prominent lesion with less-staining or abnormal stain, which contributes to detect early BSCCE.

According to the Japanese guidelines for diagnosis and treatment of esophageal cancer [15], endoscopic resection is a sufficiently radical treatment for these lesions within the mucosal epithelium or the mucosal lamina propria. Lesions reaching the muscularis mucosae or slightly infiltrating the submucosa are relative indications of endoscopic resection. Lesions exceeding the muscularis propria should be treated in the same manner as advanced carcinomas. The lesion of this case was within the mucosal lamina propria, we selected ESD as therapeutic approach because ESD has a superiority of en bloc resection of lesion which contributes to histopathologic evaluation.

In conclusions, the common features of early BSCCE in endoscopy need studies involving more patients. The key for the early detection of BSCCE remains meticulous esophagogastroduodenoscopy. The prominent lesion with a gentle rising slope and less-staining or abnormal stain might be initial feature of early BSCCE. This is a case report of the earliest BSCCE reported to date.

## Data Availability

All data and material generated or analysed during this study are included in this published article.

## Abbreviations

BSCCE: Basaloid squamous cell carcinoma of the esophagus
ESD: Endoscopic submucosal dissection
ME-NBI: Magnifying endoscopy with narrow-band imaging
NBI: Narrow-band imaging
